# Nanocurcumin Ameliorates Scopolamine‐Induced Memory Impairment by Modulating Hippocampal Akt and GSK‐3β Signaling in Mice

**DOI:** 10.1002/alz70855_097132

**Published:** 2025-12-23

**Authors:** Samaneh Reiszadeh Jahromi, Zahra Keikhosravi, Maryam Moosavi

**Affiliations:** ^1^ University of Sistan and Balouchestan, Zahedan, Zahedan, Iran (Islamic Republic of); ^2^ Shiraz University of Medical Sciences, Shiraz, Fars, Iran (Islamic Republic of); ^3^ Nanomedicine and Nanobiology Research Center, Shiraz University of Medical sciences, Shiraz, Iran, Shiraz, Fars, Iran

## Abstract

**Background:**

Memory impairment is a debilitating condition affecting millions worldwide, particularly the elderly. Alzheimer's disease, characterized by amyloid plaques and neurofibrillary tangles, is a major cause of dementia. Scopolamine, a muscarinic acetylcholine receptor antagonist, is commonly used to induce memory deficits in animal models. Curcumin, a natural polyphenol found in turmeric, has shown promise in improving cognitive function. In this study, we investigated the neuroprotective effects of nanocurcumin against scopolamine‐induced memory impairment and its underlying mechanisms. The PI3K/Akt/GSK‐3β signaling pathway plays a crucial role in neuronal survival and memory.

**Method:**

Adult male mice were randomly assigned to different groups. Memory function was assessed using novel object recognition and Y‐maze tasks. Western blot analysis was used to evaluate the expression levels of phosphorylated Akt and GSK‐3β in the hippocampus.

**Result:**

Scopolamine administration induced memory impairment, while pretreatment with nanocurcumin (2.5 mg/kg) prevented this deficit. Furthermore, scopolamine significantly decreased the ratio of phosphorylated to total Akt and reduced the phosphorylation of GSK‐3β. Nanocurcumin treatment significantly reversed these effects. In contrast, natural curcumin at the same dose did not show any protective effects.

**Conclusion:**

Our findings suggest that nanocurcumin can effectively ameliorate scopolamine‐induced memory impairment by activating the Akt/GSK‐3β signaling pathway in the hippocampus. This study highlights the potential of nanocurcumin as a therapeutic agent for cognitive disorders.

